# Piezoelectric shaping of radical mastoid cavity and dissection of ossified cochlea prior to cochlear implantation

**DOI:** 10.1093/jscr/rjag653

**Published:** 2026-08-14

**Authors:** Samiah S Al-Angari, Marc Bloching, Katharina Stölzel, Stefan Lyutenski

**Affiliations:** Department of Otolaryngology – Head and Neck Surgery, Helios Klinikum Berlin-Buch, Schwanebecker Chaussee 50, Berlin, 13125, Germany; Department of Otolaryngology – Head and Neck Surgery, College of Medicine, King Saud University, King Abdulaziz Road, AlMalaz, Riyadh, 11545, Saudi Arabia; Department of Otolaryngology – Head and Neck Surgery, Helios Klinikum Berlin-Buch, Schwanebecker Chaussee 50, Berlin, 13125, Germany; Department of Otolaryngology – Head and Neck Surgery, Helios Klinikum Berlin-Buch, Schwanebecker Chaussee 50, Berlin, 13125, Germany; Medical School Berlin, Hochschule für Gesundheit und Medizin, Rüdesheimer Straße 50, 14197, Berlin, Germany; Department of Otolaryngology – Head and Neck Surgery, Helios Klinikum Berlin-Buch, Schwanebecker Chaussee 50, Berlin, 13125, Germany

**Keywords:** piezoelectric surgery, cochlear implantation, osteoplasty, ear surgery

## Abstract

Cochlear implantation (CI) in radical cavities remains challenging due to the risk of postoperative electrode extrusion. We report a single-stage approach employing piezoelectric instrument (PEI) to optimize cavity osteoplasty, atraumatic cochlear access, and electrode stabilization in the setting of partial scala tympani ossification. A 72-year-old female with left profound sensorineural hearing loss and prior canal wall (CW)-down mastoidectomy underwent CI with posterior CW reconstruction. Angled and straight PEI inserts facilitated controlled bone removal over the facial nerve and creation of a bony slit with overhang to secure the electrode lead. Ossified segments of the scala tympani were carefully cleared, enabling full insertion of a perimodiolar electrode array, subsequently reinforced with double-layer cartilage. At 8-month follow-up, no clinical or electrophysiological evidence of electrode migration was observed. PEI is a promising tool for safe, atraumatic osteoplasty and stable electrode positioning in complex radical cavities.

## Introduction

Cochlear implantation (CI) in radical mastoid cavities is surgically challenging. There are two generally accepted surgical techniques to achieve this [1]. The first is the two-stage approach, known as subtotal petrosectomy, in which the radical cavity is completely de-epithelialized and obliterated with abdominal fat after closure of the external auditory canal. The second is a single-stage approach, in which the only additional surgical step involves reconstruction of the posterior canal wall (CW) to separate the mastoid cavity and protect the CI electrode lead.

Although the latter, in our view, provides a better option for multimorbid patients due to its shorter operating time, it nonetheless carries a significant risk of CI electrode lead extrusion [2]. In the following case, we describe a successful application of piezoelectric instrument (PEI) to reduce this risk.

## Case presentation

A 72-year-old female patient was referred for hearing rehabilitation. The left ear had a 21-year history of profound sensorineural hearing loss following CW-down mastoidectomy for cholesteatoma performed externally. The right ear showed progressive severe-to-profound mixed hearing loss after multiple external cholesteatoma surgeries, the most recent being tympanoplasty with radical mastoidectomy performed three years ago at another centre.

**Figure 1:**
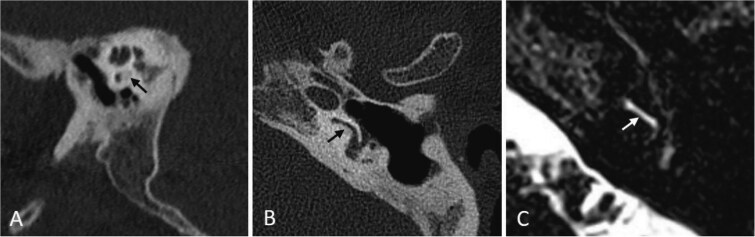
Ossified scala tympani in the basal turn of the left cochlea shown on sagittal (A) and axial (B) sections of high-resolution CT (black arrows). (C) On T2 sequences of MRI scan the ossification is causing missing hyperintensity under the scala vestibuli (white arrow).

The otologic examination showed an external auditory canal that was bilaterally wide and normal-appearing with dry radical cavities. The right tympanic membrane showed an extensive atelectasis extending to the stapes superstructure and the oval window, while the left was grey and intact. No cholesteatoma was evident bilaterally. Imaging including: computer tomography (CT) and magnetic resonance imaging (MRI) showed an ossification of the scala tympani of the inferior basal turn of the left cochlea that approximately measured 5.2 mm (Fig. 1A–C). Surgery on the right side was ruled out due to the high risk of loss of residual hearing. In light of the history, examination, anaesthesia risk, and CI candidacy criteria, we opted for a one-stage left-sided CI with reconstruction of the CW.

### Surgical technique and outcome

**Figure 2:**
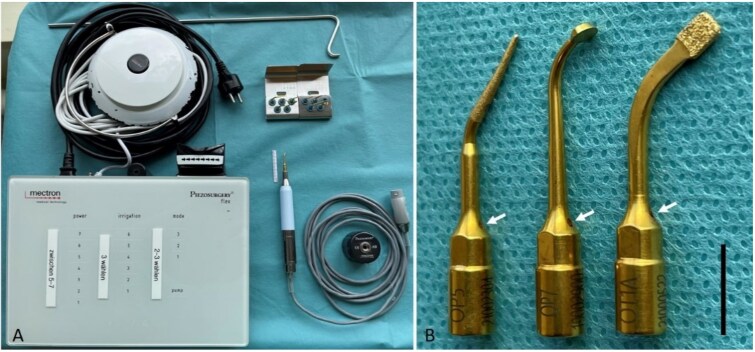
PEI (Piezosurgery flex®, Mectron, Italy). (A) Control unit with integrated peristaltic pump, foot switch, torque wrench for changing inserts, Handpiece. (B) Autoclavable inserts coated with titanium nitride used in our case series. White arrow: Exit point of rinsing solution from attachment. Scale bar: 1 cm.

**Figure 3:**
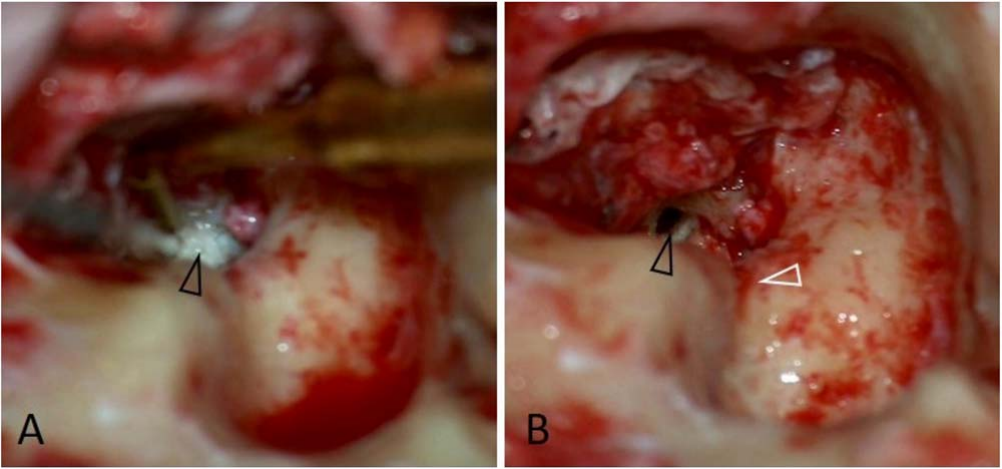
The round window niche (black arrowhead) was ossified (A). Compared to the normal bone, the sclerotic newly formed bone in scala tympani is whitish. It was followed with slightly angled awl-shaped insert (OP5), microfractured and rinsed out (B). The bony slit for the electrode slit over the remnants of the CW. The bone canal of the mastoid is clearly visible in trajectory to the opened round window (B, white arrowhead).

**Figure 4:**
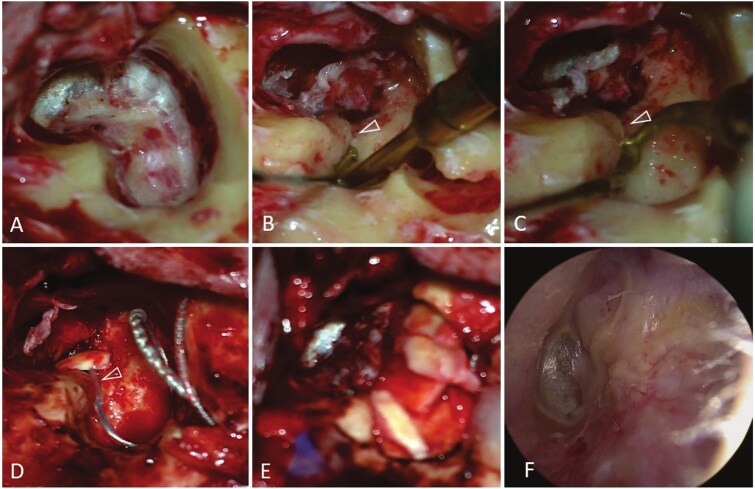
Shaping of the tympanomastoid cavity (A) and reconstruction of the posterior CW. Bony slit with a bony overhang above the facial nerve was made by using OT1A (B) and OP7 (C) inserts. The electrode lead was locked and covered by cartilage graft (D). The CW was reconstructed with Conchal cartilage (E). The healed external auditory canal was documented eight months post-operatively (F).

Under general anaesthesia, a retroauricular incision and periosteal elevation were performed. Following dissection of the epithelial lining of the radical cavity, a tympanomeatal flap was raised to expose the middle ear. The round window niche was localized and freed from scar tissue. The whitish sclerotic bone of the round window niche was removed with PEI (Piezosurgery flex®, Mectron, Italy) (Fig. 2A and B) and followed into the ossified scala tympani with awl-shaped diamond-coated insert (OP5). The contrast to the yellowish otic capsule served as an orientation (Fig. 3A and B). Linear micro-oscillations and continuous irrigation from the insert maintained a clear surgical field. In the remnants of the CW over the facial nerve, a bony slit with an overhang was constructed with curette (OP7) and wide diamond-coated grinder (OT1A) to secure the electrode lead (Fig. 4A–C). Such inserts enhanced adaptability to the mastoid anatomy. Successful cochlear access was confirmed using a probe electrode array, followed by complete insertion of the CI612 electrode (Cochlear Ltd., Sydney, Australia). The electrode lead was fixed within a prepared bony slit, enabling the first loop to lie along the mastoid cavity floor and the second to rest tension-free posterior to the reconstructed CW (Fig. 4D). Final reconstruction involved using conchal cartilage and perichondrium, providing double-layer cartilage protection, over which the epithelial lining was repositioned (Fig. 4E). The reference electrode was placed beneath the temporalis muscle and reinforced with cartilage. Demonstration of the technique is available in Video 1 in the online supplemental material.

No facial nerve injury occurred. The correct electrode array position was confirmed on the postoperative CT scan. At 8-month follow-up, the reconstructed CW was intact and stable (Fig. 4F). There were no signs of electrode dislocation in the electrophysiological measurements, with a Freiburg Monosyllabic Test of 30% under quiet conditions with CI only [3].

## Discussion

This report presents a novel surgical technique using the PEI to safely shape a radical mastoid cavity while constructing bony overhangs to secure the electrode lead during CI. To prevent electrode extrusion, adequate cartilage coverage and stable fixation are required. A critical step is drilling a bony slit in the CW remnants over the facial nerve. Because of the variable anatomical course of its mastoid segment [4], it is difficult to predict the thickness of the overlying bone. Orientation is further hindered by the absence of key landmarks, such as the incus, and by new bone formation in longstanding radical cavities. However, to construct a tight bony slit over the facial nerve, dura, and sigmoid sinus, they should not be widely exposed. Drilling in a narrow groove over the underlaying soft tissue with a conventional diamond burr carries a substantial risk of injury.

Compared with the conventional drill, the PEI is safer at the bone-soft tissue interface, particularly when the surgical view is limited [5]. Bone removal in close proximity to soft tissue with minimal contact is possible, due to cavitation induced by the insert’s linear micro-oscillations. The characteristics of the PEI could reduce the risk for injury during accidental contact with the underlaying soft tissue.

A further novel application of the PEI in this case was the removal of ossification from the round window niche and scala tympani. In our experience, conventional drills smear bone dust within the lumen during drill-out of an obliterated cochlea, obstructing the distal scala tympani and increasing the risk of modiolus injury. In contrast, PEI offers a safer and more precise alternative. The micro-oscillatory action of the PEI tip produces controlled microfractures in sclerotic cochlear bone, while continuous irrigation prevents accumulation or smearing of bone dust. This ensures clear visualization of underlying structures and facilitates precise bone removal within the ossified scala.

## Conclusion

In this case, piezoelectric surgery allowed safe shaping of the mastoid cavity and remnants of the posterior CW, as well as precise opening of an ossified cochlea. Angled inserts protect critical structures and facilitate electrode cable placement, while micro-oscillations with irrigation prevent bone dust smearing and maintain optimal visualization. Fixation of the electrode lead in the radical cavity may reduce the risk for its extrusion.

## Supplementary Material

AlangariMastoidshapingFinalwo_rjag653
